# Designing Regulatory Frameworks for Access to Genetic Resources: A Multi-Stakeholder Multi-Criteria Approach

**DOI:** 10.3389/fgene.2020.549836

**Published:** 2020-12-03

**Authors:** Aysegul Sirakaya, Klaas De Brucker, Thomas Vanagt

**Affiliations:** ^1^Faculty of Law, Department of European, Public and International Law, UGent – Ghent University, Ghent, Belgium; ^2^ABSint – Access and Benefit-Sharing International, Bruges, Belgium; ^3^Faculty of Economics and Business, Centre for Economics and Corporate Sustainability (ECON-CEDON), KU Leuven – University of Leuven, Brussels, Belgium

**Keywords:** access and benefit sharing agreements, genetic resources, multi-criteria analysis, stakeholder analysis

## Abstract

In this paper we conduct a multi-criteria analysis (MCA) from a multiple stakeholder point of view for the design of access and benefit sharing (ABS) agreements concerning genetic resources, in particular regarding the access component of such agreements. We start by analyzing the objectives defined by international law (viz. the Convention on Biodiversity and the Nagoya Protocol) that every party (i.e., all United Nations member states, except the United States) must strive to attain when developing national legislation on ABS. As countries have a certain degree of freedom concerning the way and the extent to which they need to integrate these objectives into their national frameworks and since stakeholders attach different levels of importance to these objectives, such an MCA will help identify those options that command the highest value added from the community of stakeholders. Consequently, those options are expected to hold the highest potential for successful implementation. The MCA performed in this paper is based on Saaty’s analytic hierarchy process (AHP) and starts from the objectives (i.e., criteria) enshrined in international law, and then proceeds by assessing the relative importance of these criteria from the point of view of four different stakeholder groups (industrial users, academic users, collections and provider countries). The alternatives to be evaluated in the MCA are based upon options discussed qualitatively in our previous study published in *Frontiers in Plant Science* (2019b). These options are now transformed into “design parameters” and are evaluated in terms of their contribution to stakeholder criteria. This evaluation is now performed in a quantitative way using MCA and is based on previous qualitative discussions with stakeholders that have been reported qualitatively in our previous paper in *Frontiers in Plant Science* referred to above. The final result of our MCA consists of pointing out which design parameters regarding access obtain the highest priority from the community of stakeholders and hence need to be present in national regulatory frameworks on ABS that will be implemented by member states. It is our intention to undertake similar research for the Benefit Sharing component of ABS agreements in the future.

## Introduction

Genetic resources (GR) constitute a crucial source of provisioning ecosystem services (The Economics of Ecosystems and Biodiversity, 2010). On top of being used within the field of academic research, GR are utilized by many industries such as agriculture, food industry, pharmaceuticals, cosmetics, energy (Laird and Wynberg, 2018).

Natural product research dates back to the 18th century when systematic explorations begun when the newly found species were appropriated by European colonial powers and shipped to Europe where they were used as herbs, food and medicine. During the colonial period, compensation schemes between the explorers and locals sharing their knowledge on the use of these species were completely non-existent (Pistorius, 1997).

The value of GR has received a second boost since the 70s from modern biotechnology. Its economic value created a strong incentive to apply patents on inventions resulting from the use of GR. Since patenting is a rather costly procedure, patents on inventions resulting from the use of GR were mostly held by companies in developed countries. With the majority of the world’s biodiversity being present in developing countries and use of these GR being mainly undertaken by companies and institutions in developed countries, the perception of imbalance thereof led to discussions on fair and equitable sharing of benefits arising from the utilization of GR (Oberthür and Rosendal, 2014).

The present system of access and benefit-sharing of GR (ABS) aims to balance the rights of provider countries (or simply “providers”) through the equitable sharing of benefits, and those of the users. Ultimately, ABS could increase the value of biodiversity, for users as a resource for R&D, as well as for providers as a source of benefits that create incentives for biodiversity conservation.

1ABS has been introduced to the international legal forum, starting from 1992 with the Convention on Biological Diversity [CBD] (1992). The most recent international legal instrument on ABS is the Nagoya Protocol on Access to Genetic Resources and the Fair and Equitable Sharing of Benefits arising from their Utilization (Nagoya Protocol, 2011), which came into force in 2014. Additionally, a specialized instrument, the International Treaty on Plant Genetic Resources for Food and Agriculture (ITPGRFA), deals with many of the core plant genetic resources to be used for breeding purposes. The multilateral system (MLS)’s legacy under the ITPGRFA is seen as jeopardized due to the fact that the Parties have not reached a consensus especially regarding the provision of the Digital Sequence Information (DSI) and the revision of the benefit-sharing rates under the Standard Material Transfer Agreement (sMTA) in their 9th *Ad Hoc* Open-Ended Working Group, which was the last one to have received a mandate. In addition, some countries already stipulated that they may regulate PGRFA under Annex I under domestic ABS measures. For instance, during the 8th Meeting of the Governing Body of the ITPGRFA in November 2019, Zambia stated that since there is no progress on benefit-sharing in the Treaty, Africa may explore regulating DSI related to PGRFA within the MLS under national ABS measures (IISD, 2019).

The provisions of the CBD originate from its overarching three-pillar objective which consists of: (1) conservation of biological diversity, (2) sustainable use of the components of biodiversity and (3) fair and equitable sharing of benefits arising from GR. Therefore, the importance of the ABS system enshrined in the Nagoya Protocol as well as the CBD is increasing for plant genetic resources under the ITPGRFA.

Article 15 of the CBD reaffirms the states’ sovereign rights over their GR. This means that states have the right to regulate access to their GR, which includes the right to determine the conditions of such access and the fair and equitable benefit-sharing resulting from the utilization of GR (Kamau and Winter, 2013).

Regarding ABS in national jurisdiction, international law by means of the CBD and the NP puts in place general goals and principles for states to implement. Currently, every United Nations Member State except for the United States is a party to the CBD (Convention on Biological Diversity [CBD], 2018). The NP on the other hand currently has 123 Parties; a number which is expected to increase (Convention on Biological Diversity [CBD], 2011).

ABS is currently a rapidly developing and evolving field that is shaped by the national implementation by the Parties concerned, which determines how ABS goals are realized and how ABS principles are formed within regulatory mechanisms.

The current perception regarding the operationalization of ABS is that the system of national implementation brings complexities for both academic and industrial users of GR as well as collections (such as natural history museums, botanical gardens) which result in less willingness to access GR (Koester, 2012; Watanabe, 2015; Lassen, 2016; Overmann and Scholz, 2016). Users commonly list the lack of legal certainty, inconsistency in ABS systems, reputational risk and investment uncertainty as the main reasons for concern (UNCTAD, 2017). Less access would inevitably result in fewer benefits shared and this vicious cycle would jeopardize the success of the entirety of the ABS system.

The overarching reason why ABS implementation is neither beneficial for the providers nor for the users is that the provider countries fail to implement an adequate institutional framework that is consistent with the international ABS goals and therefore implementation does not fulfill the objectives of the international ABS framework. This inevitably jeopardizes access to GR as well as benefit-sharing and thus damages trust between the provider and the user.

## Aim of This Paper and Methodology Applied

### Aim of This Paper

Our previous research (Sirakaya, 2019a,b) aimed at identifying and evaluating international ABS goals and regulatory options that have the highest potential to create an adequate institutional framework able to address the above-mentioned problem. In Sirakaya (2019a) we identified and analyzed international goals that establish the general principles of the international ABS frameworks. In Sirakaya (2019b) we took an evaluative approach by subjecting the identified common regulatory options to stakeholder interviews. Our present research reported here aims to identify stakeholder opinions regarding the current ABS framework and to design novel types of ABS framework that better fit with the preferences of the stakeholders involved and that are at the same time in line with international ABS goals. Consequently, the novel types of ABS frameworks designed in this way will be associated with an increased chance of successful implementation.

### Methodology Used in This Paper

#### Stakeholder-Driven MCA

To address the research objectives defined in section “Aim of This Paper” we will conduct a multi-criteria analysis (MCA). This MCA will be designed so that it can play the role of an “institution in action” as described in De Brucker et al. (2013). Institutions can be defined as “the rules of the game in a society” (North, 1990:3) or as “decision procedures,” i.e., a set of rules that enable a group or society to transform individual (or stakeholder) preferences into collective preferences. The essence of economics is – according to Commons (1934/1959) traditional institutionalist definition – to solve or at least manage social conflicts so as to increase economic welfare. Here, conflict among stakeholders’ interests may enhance creativity, value-focused thinking (Keeney, 1996) and constructivism, i.e., the act of looking for better or more efficient solutions. It is from the clash of ideas that true insight can spring (cf. the quote by French writer Boileau, 1636–1711, “*du choc des idées jaillit la lumière*”). MCA can be viewed as an institution in action creating momentum to achieve this goal.

Hence, stakeholders will play a crucial role in this MCA. According to Freeman (1984), stakeholders are defined as “any individual or group who can affect an organization’s performance or who is affected by the achievement of this organization’s objectives.” The idea of integrating stakeholder points of view in an MCA was first developed by Macharis (2005) and Macharis et al. (2007) through so-called “multi-actor multi-criteria analysis” (MAMCA), and the idea of using it as a tool for intentional design (following a two-step procedure) to evolve institutional frameworks or implement new technology came from De Brucker et al. (2011, 2013, 2014, 2015).

Looking into regulatory issues related to ABS, we have identified four key stakeholders: the government (as the provider), collections, academic users and industrial users. These have been identified in line with Freeman’s definition. These key stakeholders’ involvement in regulatory processes is vital to forming an ABS system that is effective and efficient and that attends to the international ABS goals (Swiderska, 2001). In other words, the stakeholders identified for this study are either the regulators or the subjects of the regulation.

#### Construction of a Value Structure Including Stakeholders, Criteria, Design Parameters

**FIGURE 1:**
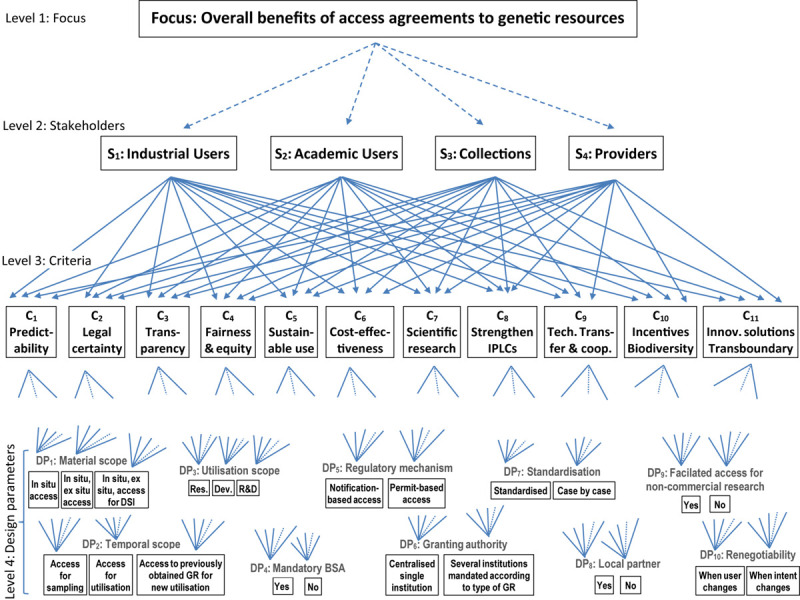
Value structure for the overall benefits to be derived from the design of access agreements to GR (Own design).

The value structure is shown in Figure 1. The top level represents the focus, i.e., the overall objective, namely generating societal benefits by designing adequate agreements to obtain access to GR.

At the second level the stakeholders (*s*) are shown. Here four main groups of stakeholders have been identified as “industrial users” (such as pharmaceutical companies, food/feed industry), “academic users” (such as universities), “collections” (such as natural history museums, botanical gardens) and “providers” or “provider states” (i.e., countries like Brazil and India that are home to a rich pool of GR). The level below the stakeholders (i.e., level 3) comprises the criteria that these stakeholders consider relevant. The degree to which these criteria are relevant for the different stakeholder groups is reflected in the weights (or priorities) of the criteria. These weights will be derived below (in section “Building Consensus Among Stakeholder Representatives’ Scorings”). The set of 11 criteria shown at level 3 of the criteria tree has been constructed based on our previous research (Sirakaya, 2019b) on identifying international ABS goals embedded in international documents on ABS such as CBD, NP and related decisions of the Conference of the Parties to both CBD and NP (i.e., COP Decisions). An analysis of these documents showed that there are 11 ABS goals that are prescribed by these documents. Hence, these goals must be fulfilled by the parties through their national ABS frameworks and the regulatory mechanisms installed in national ABS frameworks must be consistent with these goals. These goals, which are listed below, have, therefore, been operationalized as the criteria shown on level 3 of Figure 1.

C_1_: Predictable conditions (NP Preamble).
C_2_: Legal certainty (NP Article 6, COP Decision V/26, VII/19, VIII/4).
C_3_: Transparency (NP Article 6, COP Decision V/26).
C_4_: Fairness and equity in negotiations (NP, COP Decision V/26).
C_5_: Sustainable use of biodiversity components (CBD Article 1, NP Preamble, Article 8, Article 9, COP Decisions V/26 and VII/19).
C_6_: Cost-effective measures (NP Article 6, COP Decisions VII/19, VIII/4).
C_7_: Scientific research based on GR (CBD Article 15.6).
C_8_: Strengthening the ability of Indigenous People and Local Communities to benefit from the use of traditional knowledge (NP Articles 5, 6, 7, 12, 21, 22,/COP Decision V/26, VI/24).
C_9_: Tech transfer and cooperation to build research and innovation capacity in developing countries (NP, COP Decisions VIII/4, VII/19 VI/24, V/26).
C_10_: Creating incentives to conserve biodiversity (CBD Article 11, COP Decision VI/24, NP Preamble).
C_11_: Innovative solutions for transboundary situations (NP Preamble and Article 11).

When developing this set of criteria special attention has been given to the technical conditions or requirements for a consistent family of criteria (Roy, 1996, pp. 216–219, Belton and Stewart, 2002, pp. 55–58. The criteria subject to this study are assumed to be exhaustive. All of them derive from international legal documents on ABS which are a result of extensive stakeholder input and consultation. Furthermore, we asked stakeholders whether more criteria should be considered, and whether any additional goals should be considered. In terms of relevance, as these criteria were internationally agreed and need to be addressed in national laws, their relevance for the regulator and the subject of the regulation is salient.

The non-redundancy of the criteria has also been checked. The authors initially identified a 12th criterion (workability/operational functionality) yet decided to drop it since the conjunction of several criteria (cost-effectiveness, legal certainty and predictability) made the 12th criterion redundant. This check also contributed to the simplicity of the criteria set.

Comprehensibility of the criteria has been ensured by subjecting it to stakeholder review where they were asked to explain what they understand from the wording of the objectives.

The criteria satisfy judgmental independence as a stakeholder’s perception of them does not depend on another criterion. For instance, a stakeholder does not exhibit preference dependence between “predictable conditions for ABS” and “transparency” meaning that the stakeholder’s perception on one will not depend on the other.

The lowest level in the hierarchy shown in Figure 1 (i.e., level 4) contains the actual regulatory options (or alternatives) to be evaluated. Here the options are to be seen as design parameters (DP). The aim of the MCA here is to assess which particular ways of designing ABS systems command the highest priority from the different stakeholders. More details about the specific features of these regulatory options are described in Sirakaya (2019b). A description follows.

For instance, regarding the material scope (DP_1_), three different options are possible, namely “*in situ* access only,” “*in situ* and *ex situ* access” and “*in situ* access, but *ex situ* access for DSI.” With “*in situ* access only,” a permit or notification is only required when access happens within the geographical borders of the provider country. In the event of “*in situ* and *ex situ* access,” a permit or notification is also required when access takes place through biodiversity biobanks (that contain biological samples) outside the geographical borders of the provider country. The third option also covers *ex situ* access to the digital sequence information (DSI), which is often referred to as the DNA sequence data of the GR that is downloadable from public databases. Here it should be noted that there currently is no unified definition of DSI both in the legal and scientific realm.

Regarding the temporal scope (DP_2_) three options are possible. With “access for sampling” the access requirements need to be triggered before the material is sampled *in situ* or obtained from an *ex situ* source. In the event of “access for utilization” the user first needs to obtain the ability to perform R&D. In the event of “access to previously utilized genetic resource for new utilization,” this requirement also needs to be triggered when a new utilization activity occurs with a genetic resource that was previously made available to the user.

As regards the utilization scope (DP_3_), the option “research” implies that access is only permitted for research purposes. With the option “development” access is permitted for product or process development purposes. Under the option “research and development” access is permitted for both purposes.

In terms of benefit sharing agreements (BSA) (DP_4_), two options are possible. The can be either mandatory or non-mandatory.

Regarding the regulatory mechanism (DP_5_), the access can be subject to either a permit or a mere notification. In the latter case the user can commence activities without having to wait for approval.

Regarding the granting authority (DP_6_), either a single centralized institution can be made responsible or several decentralized institutions can be mandated according to the type of GR.

Regarding standardization (DP_7_), the provider country can either opt for standardizing the access procedure or apply various conditions on a case-by-case basis.

Making an agreement with a local partner (DP_8_) can either be a prior requirement or not.

Furthermore, access for non-commercial research (DP_9_) can be facilitated or not.

And finally, the access agreement may be subject to renegotiation (DP_10_) when the user changes or when the intent changes.

Consequently, at this stage of the analysis, it is these options (i.e., the options within the different DPs) that need to be evaluated and prioritized. Hence, comparisons or prioritizations of combinations of options across DPs (e.g., comparing “*in situ* access” to e.g., “access for utilization”) are not made at this stage.

#### The Choice of the AHP-MCA

The actual MCA technique will that will be used to obtain the priorities of the criteria and finally also those of the alternatives (regulatory options, or in MCA terminology; design parameters) is the analytic hierarchy process (AHP) of Saaty (1977, 1986, 1988, 1995). The AHP method has been chosen for several reasons. The first reason is that AHP makes it possible to decompose a complex decision-making problem into its constituent parts. When treated separately these often are less complex to manage. The hierarchy shown in Figure 1 visualizes how the DM problem has been decomposed into its constituent parts. Each arrow in Figure 1 represents a causal relationship. For instance, the 11 arrows that point from the stakeholder “Industrial users” (on level 2) to the 11 criteria (on level 3) indicate that these criteria (or more precisely the scores obtained by the alternatives on those criteria) contribute to the objectives of the stakeholder “Industrial users.” Likewise, the three arrows that link the criterion “Predictability” (C_1_) with the three options for design parameter “Material scope” (DP1), namely “*in situ* access,” “*in situ*, *ex situ* access” and “*in situ*, *ex situ* access for DSI” also represent causal relationships in the sense that each of the latter options contributes to the realization of the criterion “Predictability.” But each option of each design parameter also contributes to each of the 11 criteria (on level 3). For instance, the 11 arrows that link “*in situ* access” with the criteria C_1_…C_11_ indicate that “*in situ* access” contributes to the realization of each of these 11 criteria. In order not to overload the figure with arrows, the latter type of arrows have not been drawn up to their full length.

Another reason why we chose the AHP is that the final decision can be constructed step by step, using an interactive procedure based on input from stakeholders, policymakers and experts. The evaluative approach followed here is clearly a constructivist one that facilitates learning during the DM process. In fact, the evaluations (pairwise comparisons) that take place within each constituent part of the hierarchy (shown in Figure 1) are synthesized using a step-by-step procedure so as to obtain a final ranking (priorities) of the options studied. This is the case not only for the calculation of the priorities of the alternatives (or here: the options for the design parameters or regulatory options) in terms of the criteria, but also for deriving the weights of the criteria. The latter are furthermore determined endogenously, based on input from stakeholders or policymakers.

Given the hierarchical decomposition of the DM problem, the priorities of the final solutions can be calculated from the perspective of each single stakeholder. The priorities obtained in this way can form the basis for the construction of a solution (in this case the design of particular types of access frameworks) that contributes to the objectives of the different stakeholders. Very important here is the degree of compatibility between the stakeholder priorities. Stakeholders are seen here as the critical (f)actors that determine whether a solution (here a type of access agreement) has the potential to be successful in practice or not. If the compatibility between stakeholder priorities is low, the potential of the solution to be accepted by all stakeholders and hence to make possible successful and sustainable access to GR may be problematic. The aim of the MCA developed here is to identify those solutions that have potential and those that have not. If the MCA shows that some solutions do not have the required potential, but that this is due to some specific reasons (as shown by the scores on the criteria), then at a second stage, the potential of these solutions may be enhanced through project redesign (De Brucker et al., 2015). This means that a better alternative may be constructed by remedying (or compensating) the weaknesses of the initial alternative or by adapting the legal or institutional framework (i.e., reducing the threats or transforming them into opportunities).

Another important advantage of the AHP-MCA is that the AHP makes it possible to take into account heterogeneous information (both quantitative and qualitative) and to integrate it into the DM process. Particularly as regards the design of ABS frameworks, a lot of the information (i.e., the scores on the criteria) will be qualitative.

Finally, the AHP makes it possible to obtain a complete ranking of all the solutions and to calculate the logical consistency of all the pairwise comparisons on which the final priorities for the solutions are based.

#### The Use of the Pairwise Comparison Mechanism in AHP (in General)

In each subsystem of the AHP (i.e., each system formed by the higher level element and all the lower level elements with which a causal relation exists) pairwise comparisons need to be made to derive the relative priorities of all the lower level elements in terms of their contribution to the higher level elements. This is done using a pairwise comparison matrix (*A*) as shown in Table 1. For each pair of elements (e.g., the actions *a*_*i*_ and *a*_*i”*_) the DM has to assign a value to the relative importance of one action (*a*_*i*_) as compared to another action (*a_*i*__’_*) in terms of its contribution to a higher level objective or criterion (*c*_*j*_), as shown in Table 1. The value *Pg*_*j*_(*a_*i*_,a_*i*__’_*) expresses the DM’s intensity of preference for the row element (i.e., the element to the extreme left of the row, in this case *a*_*i*_) as compared to the column element (i.e., the element on top of the column, in this case *a_*i*__’_*) in terms of its contribution to the higher level element (i.e., the element mentioned in cell at the intersection of the first column and the first row of the matrix, in this case the criterion *c*_*j*_). This procedure is repeated at each level of the hierarchy, and hence is used for the prioritization of the actions (i.e., comparing the actions in pairs in terms of their contribution to the criteria to which they contribute) as well for the prioritization or weighting of the criteria (i.e., comparing the criteria in pairs in terms of their contribution the higher level stakeholder’s objectives). A computer program called ExpertChoice can be used for this purpose.

**TABLE 1:** Pairwise comparison mechanism in the AHP.

**c_j_**	**a_1_**	**…**	**a_i__’_**	**…**	**a_n_**
**a_1_**	1				
**…**		[1]			
**a_i_**			Pg_j_(a_i_,a_i__’_)		
**…**				[1]	
**a_n_**					1

The intensities of preference, i.e., the values *Pg*_*j*_(*a_*i*_,a_*i*__’_*), are expressed on a fundamental scale (i.e., the so-called Saaty scale), ranging from 1 to 9 (Saaty, 1995, p. 73). A qualitative (semantic) label is associated with each value on this scale as shown in Table 2. The values from this scale may also be conceived as estimates of ratios (even for intangible effects).

**TABLE 2:** Pairwise comparison scale in the AHP (Source: Saaty, 1995, p. 73).

**Intensity of** **importance** **Pg_j_(a_i_,a_i’_)**	**Definition**	**Explanation**
1	Both elements have equal importance	Both elements contribute equally to the criterion considered
3	Moderately higher importance of row element (RE) as compared to column element (CE)	Experience and judgment reveal a slight preference of row element (RE) over column element (CE)
5	Higher importance of RE as compared to CE	Experience and judgment reveal a strong preference of RE over CE
7	Much higher importance of RE as compared to CE	RE is very strongly favored over CE, and its dominance has been demonstrated in practice
9		Complete dominance in terms of importance of RE over CE	The evidence favoring RE over CE is of the highest possible order
**2, 4, 6, 8** (Intermediate values)		Intermediate position between two assessments
**1/2, 1/3, 1/4,…1/9** (reciprocals)		When CE is compared with RE, it receives the reciprocal value of the RE/CE comparison
**Rationals** Ratios arising from the scale		If consistency were to be forced by obtaining *n* numerical values to span the matrix
**1.1–1.9** For tied activities		RE and CE are nearly indistinguishable; moderate is 1.3 and extreme is 1.9

On the basis of the numerical information associated with these statements, relative priorities or weights are calculated using the eigenvector method. Mathematically the relative priorities, i.e., the weight vector (*W*), is given as the right eigenvector (*W*) corresponding to the highest eigenvalue (*λ_*max*_*) as shown in Eq. 1. The matrix *A* (in Eq. 1) is the matrix containing all the pairwise comparisons and corresponds with Table 1.

(1) $$A\cdot W=\lambda_{\max}\cdot W$$

In order to synthesize all local priorities, the various priority vectors (*W*) are weighted according to the global priorities of the parent criteria and synthesized. One starts from the top and by doing so, the final/global relative priorities for the lowest level elements (i.e., the actions) are obtained.

In each matrix, a number of pairwise comparisons are redundant, since the preference intensity of action *a* compared to action *c* (in terms of their contribution to criterion *g*_*j*_) should, in the event of complete consistency of all pairwise comparisons within the same matrix, equal the product of the preference intensities of action *a* compared to action *b* and the preference intensity of action *b* compared to *c*, as shown by Eq. 2.

(2) Pg(a,c)j=Pg(a,b)j⋅Pg(b,c)j

However, this redundancy is used for two purposes, namely to neutralize estimation errors that may have occurred in other pairwise comparisons of the same matrix (in fact information is being accumulated using multiple/redundant measurements) and to check the consistency of all pairwise comparisons within one matrix. According to Saaty (1985, p. 81) a limited amount of inconsistency is quite natural and does not pose a problem as long as the inconsistency ratio (ICR) does not exceed 10% (i.e., 0.10).

In some cases, the number of pairwise comparisons to be made in the AHP may become quite large. However, when for instance regarding the evaluation of the actions in terms of the higher-level criteria, information or scores expressed on a ratio scale that has the properties of a cardinal value function is available (or can be constructed), then this information can be used directly to derive the relative priorities. In that case pairwise comparisons are not necessary for that part or subsystem of the hierarchy. When an underlying cardinal value function exists, this means for instance that an action that obtains a score x times as high on this scale as another action is also considered to be associated with a utility (or value) level that is also x times as high as the other action. This can be the case for instance for a criterion like “public expenditure.” A project that requires a level of public expenditure e.g., three times as low as another project can be considered to be three times as good (in terms of utility) as the other project. Not all ratio scales possess this property. For instance, as far as the criterion “salary” is concerned, a candidate evaluating different job offers will not necessarily consider a job paying a monthly salary of €6,000 to be twice as good as a job with a salary of €3,000, *ceteris paribus*. The reason is that there is decreasing marginal utility of income for an individual. In the former example (public expenditure) utility of income may not be decreasing because of the societal perspective taken. Also ordinal scales such as ++/+/0/−/−− do not necessarily possess this utility. This means that in that case pairwise comparisons will be necessary. However, some techniques can be used to transform these scales into ratio scales with underlying cardinal value properties, for instance by making the pairwise comparisons in terms of value or utility levels only once, but at the level of the score categories (as will be explained below).

#### Evaluation of ABS Systems Based on Pairwise AHP Comparisons

##### General

In order to obtain relative priorities (weights) for the criteria shown on level 3 of the hierarchy (in Figure 1), stakeholder representatives have been surveyed.

Each group of stakeholders (academic users, industrial users, providers countries and collections) were asked to rank on a five-point scale the international ABS goals explained in section “Conclusions” of this paper, according to the perceived impact on their daily operations with regards to GR. This survey was conducted between March 13, 2018 and April 30, 2018 and was sent to over 600 stakeholders including all of the national competent authorities of parties to the CBD, all of the national focal points, academic institutions, collections and industrial users worldwide. The selection of the stakeholders is based on their role in their institution as well as their demonstrated interest in ABS (published articles, their position and expertise, attendance at conferences, workshops or discussions related to ABS). The survey has received 220 responses in total with 92 responses from providers, 60 from academic users, 31 from industrial users and 37 from collections. Even though the number of participants is not homogenously divided between the stakeholder groups, the answers of a strongly represented stakeholder group (e.g., provider countries) did not have an impact on the overall result in comparison to a group with fewer responses. This is because four individual surveys were created for each stakeholder group (e.g., academic users).

The survey is designed as separate blocks for each stakeholder in order to ease the process of delineating and processing data into units (Elliott and Timulak, 2005). It consists of four questions for each stakeholder group, two of them asking various stakeholders to rank the importance of international ABS goals. Questions 1 and 2 ask stakeholders to rank the importance of international ABS goals for “access” (question 1) and “benefit-sharing” (question 2) to GR on a scale of 1 to 5 by means of qualitative categorizing (very important, important, no effect, unimportant, very unimportant). Question 3 asks whether the stakeholder thinks more goals should be included at the international level. Question 4 asks if the stakeholder is available for an in-depth interview. The qualitative indication of importance on a five-point scale assists this study in defining stakeholders’ priority setting per ABS goal.

The survey did not request the names or contact details of the stakeholders except for question 4 in order to preserve anonymity to avoid socially desirable answers (Gosen, 2014).

The stakeholder interview has been designed in a semi-structured manner. The questions on access and compliance asked stakeholders to rank their preference per regulatory option on a scale of 1 to 3 with 1 being the most favorable and 3 being the least favorable. The questions on benefit-sharing asked the stakeholders to rank the impact (from very positive to very negative) and burden (from burden to very heavy burden) of engaging in the given monetary or non-monetary option.

As the number criteria is quite high (11), the number of pairwise comparisons to be made becomes quite large as well. In an 11 by 11 matrix a total of 55 pairwise comparisons may need to be made (for the diagonal and the lower triangle of the matrix no pairwise comparisons need to be made as the lower triangle values are the reciprocal of the upper triangle values and the diagonal line refers to comparisons between identical elements, which always have value 1). In addition, the number of stakeholder representatives is also quite large. Therefore, one of the techniques referred to in section “The Use of the Pairwise Comparison Mechanism in AHP (in General)” to obtain a cardinal value function has been used. To derive the criterion weights, the various stakeholder representatives were asked to score the importance of the criteria to them on a five-point ordinal scale (viz.++/+/0/−/−−), whereby “++” corresponds to “very important,” “+” to “important,” “0” to “no effect or neither important, nor unimportant,” “−” to “low importance” and “−−” to “very low importance.” To make possible ratio scale comparisons and derive AHP-like relative priorities, this ordinal scale has been transformed into a ratio scale using pairwise comparisons at the level of the score categories. This is shown in Table 3, Part A. This needs to be done only once, namely at the level of the score categories and not for each pair of criteria. This technique reduces the number of pairwise comparisons to be made drastically.

**TABLE 3:** Pairwise comparison of score categories and corresponding ratio values.

**Part A: Pairwise comparison of score categories**						**Part B: Corresponding ratio values**	
**S_k_**	**++**	**+**	**0**	**–**	**– –**	**S_k_**	**Ratio value scale**
**++**	1	3	5	7	9	**++**	0.527
**+**		1	2	5	7	**+**	0.249
**0**			1	2	3	**0**	0.118
**–**				1	2	**–**	0.065
**– –**					1	**– –**	0.041
IC = 0.02							

For instance, the value 7 (cell shaded in Table 3, Part A) expresses the idea that the element to the left of the row, in short the “row element” (++) conveys a “much higher importance” than the element on top of the column, in short the column element (–). This means that when an element that scores “++” on the ordinal scale used in the survey is compared to an element that scores “–” in the same survey, the former element is considered to be five times more preferable than the latter.

The values in Table 3 (Part A) have been carefully selected. When two of the lower score categories that are close to each other are compared (e.g., “–” and “– –”) a low value on the Saaty scale was given (score 2, which represents a hesitation or compromise between “equal importance” and “moderately higher importance”). But when two adjacent scores of the higher score categories (e.g., “++” and “+”) are compared a slightly higher value on the Saaty scale was given (score 3, i.e., “slightly higher importance”). The score difference between “++” and “+” is assumed to be higher than the score difference between “–” and “– –.” When two extreme scores are compared (e.g., “++” and “– –”), then a very high value on the Saaty scale was given (value 9 corresponding with an “extreme dominance” of the row element over the column element).

The ratio value scale comparisons that result from this comparison at the level of the score categories (++,+, 0, –, – –) are calculated as the right eigenvector corresponding to the highest eigenvalue as outlined in Eq. 1. The results are presented in Table 3, Part B.

##### Building consensus among stakeholder representatives’ scorings

Since we surveyed quite a large number of representatives for each stakeholder, the scores (or importance levels) for the criteria as expressed on the five-point ordinal scale referred to above differ from each other to some extent. The ideal situation is that the various stakeholder representatives would achieve a consensus on the final importance level to be given to the 11 criteria. Such a consensus might be obtained when all these representatives gathered for a meeting. But since the number of representatives per stakeholders was quite high and these representatives come from different countries all over the world (ranging from Brazil to Japan and from Norway to South Africa) we had to follow a more pragmatic procedure to build the consensus score. This procedure is based on statistical measures like the median and is illustrated in Table 4.

**TABLE 4:** Overview of responses by stakeholder representatives.

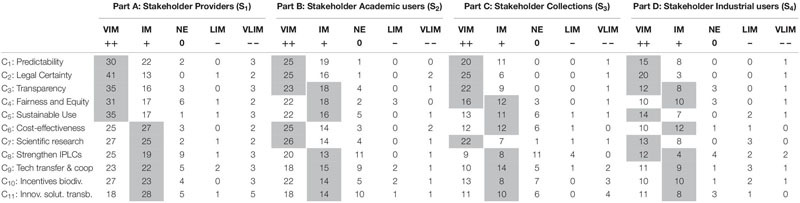

*VIM, Very Important; IM, IMportant; NE, No Effect, neither important nor unimportant; LIM, Low IMportance, VLIM, Very Low Importance.*

The cells in Table 4 (except those on the first row and first column) represent the number of stakeholder representatives that assigned the respective score category to the importance level of the respective criteria. For instance, from a total of 57 representatives from the stakeholder “Providers” that gave a score to the criterion “Predictability” (C_1_), 30 representatives placed this criterion in the category “very important,” 22 placed it in the category “important,” 2 placed it in the category “no effect” and no representative placed it in the category “low importance” and 3 representatives placed it in the category “very low importance” (see Table 4, Part A, first row below header row). The score category that contains the median value has been shaded in Table 4. For instance, as regards C_1_ (Predictability) scored by 57 representatives from the stakeholder “Providers” (i.e., Part A of Table 4), the middle value (i.e., the median) corresponds to the 29th score (when ordered) and this score falls within the category “very important.”

The calculation of the criteria priorities or weights in the AHP has thus been based on the median value. The idea behind this is that if all stakeholder representatives had to come together in a meeting to obtain a consensus on the final score category to assign to the importance of the criteria (e.g., using a voting procedure), the consensus solution that would have emerged out of this meeting would be very close to the median score. If necessary, a sensitivity analysis will be performed where we will test to what extent the final ranking of the final alternative solutions might change when other weight sets were used.

The final relative priorities for the criteria are shown in Table 5 for the four stakeholders. These are based on the median value of the stakeholder representatives’ scores. For instance, as regards the stakeholder “Providers,” the median value of stakeholder representatives’ scores for C_1_ to C_5_ is “very important” (see Table 4, Part A) and – based on pairwise comparisons at the level of the score categories (Table 3, Part A) – the category level “very important” obtained a relative priority of 0.527 in Table 3, Part B. For the criteria C_6_ to C_11_ the median value (in Table 4, Part A) is “important” which corresponds with a relative priority of 0.249 (in Table 3B). These values were finally normalized (i.e., divided by their total sum, which is 5 × 0.527 + 6 × 0.249 = 4.129). Consequently, the relative priority of C_1_ (and also C_2_…C_5_) in Table 5 (Part A) is 0.527/4.129 = 0.128 and the relative priority of C_6_ (and also C_7_…C_11_) is 0.249/4.129 = 0.060.

**TABLE 5:** Relative priorities (weights) for criteria regarding Access from four separate Stakeholders’ Perspectives.

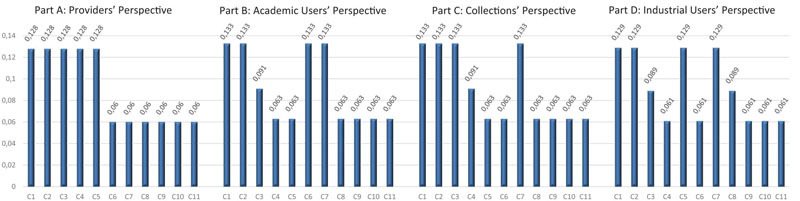

*C_1_: Predictable conditions; C_2_: Legal certainty; C_3_: Transparency; C_4_: Fairness and equity in negotiations; C_5_: Sustainable use of biodiversity components; C_6_: Cost-effective measures. C_7_: Scientific research based on GR; C_8_: Strengthening the ability of Indigenous People and Local Communities to benefit from the use of traditional knowledge. C_9_: Tech transfer and cooperation to build research and innovation capacity in developing countries; C_10_: Creating incentives to conserve biodiversity. C_11_: Innovative solutions for transboundary situations.*

However, in some cases, e.g., when two scores share the middle position in the stakeholder representatives’ rankings, a compromise has been made. For instance, as regards the criterion “Transparency” (C_3_) for the stakeholder “Academic users” (in Table 4B), there is an even number of scores (46) and in that case both the 23th and the 24th score share the middle position. However, the former falls within the category “very important” and the latter within the category “important.” In this case the geometric mean of both the relative priorities associated with these score categories (from Table 3B) was given to this score, namely (0.527 × 0.249)^1/2^ = 0.362 and this result was then used as input into the normalization process described above but applied to the stakeholder “Academic users.” Hence, the criterion C_3_ obtains, from Academic users’ perspective, a normalized relative priority of 0.362/(0.527 × 4 + 0.362 × 1 + 0.249 × 6) = 0.091. The geometric mean was used here (as suggested by Saaty, 1995, pp. 265) as we are calculating the average of ratios.

##### Prioritization of the design parameters

The prioritization of the design parameters in terms of the criteria to which they contribute (as visualized in Figure 1) was done in a comparable way to the prioritization of the criteria in terms of the stakeholders’ objectives. The difference, however, is that the evaluations of the options for the design parameters were not based on a large survey, but directly based on expert judgment. To this end, an expert rated the options in terms of their contribution to the criteria considered relevant by the stakeholders.

The stakeholder survey conducted in Sirakaya (2019a) included a question on the participant’s availability for an in-depth interview regarding ABS options. A total of 53 of the 220 respondents demonstrated their interest and 20 ended up participating to the interview. The distribution of the participants amongst the stakeholder groups has proven to be rather homogenous as five experts represented provider countries, six experts represented collections, five represented industrial users and four represented academic users. Written informed consent forms were obtained from all of these experts.

The analysis and scoring of the interview results derive from both the literature review in Sirakaya (2019a) and the interview analysis in Sirakaya (2019b). Since the interviewees were initially the respondents to the surveys related to ABS goals, their perception of the effect of the regulatory options on the goals has not been difficult to obtain. Where the number of responses was not sufficient, an additional literature review has been conducted with the aim of aiding the scoring phase.

For instance, when scoring stakeholders’ views on DSI in the light of ABS criterion N°7 (scientific research on GR), we have utilized much of the related literature (Vogel, 2011; Bagley and Rai, 2013; Lawson and Rourke, 2016; Devi and Pisupati, 2018; Wynberg and Laird, 2018; Flach et al., 2019; Geary and Bubela, 2019; Watanabe, 2019) to fine-tune our conclusions about how regulating DSI in the same manner as GR would negatively impact scientific research activities including those conducted in provider countries. The fact is that providing access in a timely manner would be challenging and track and trace would be inefficient for both the users and providers.

Likewise, when refining our conclusions related to the temporal scope, as well as the activity scope, and the implications on criteria N°1 (predictability), N°2 (legal certainty) and N°3 (transparency) thereof, we resorted to many sources of literature (Seiler and Dutfield, 2001; Orsini et al., 2008; Andersen et al., 2010; Schei and Tvedt, 2010; Oliva, 2011; Coolsaet and Pitseys, 2014; Morgera et al., 2014; Prip and Rosendal, 2015; Wyss, 2017) in order to be able to rank the best option to enable the attainment of these goals. The diversity in our literature sources allowed us to enrich our conclusions regarding stakeholder opinion.

The scores that finally resulted from the procedure described above and that express the contribution of the different options within each single DP (like DP_1__A_, DP_1__B_, DP_1__C_; DP_2__A_, …, DP_10__B_) to the 11 criteria (C_1_,…,C_11_) are given in Table 6.

**TABLE 6:** Relative priorities (weights) for the criteria the four stakeholder categories.

	**C_1_ Predictability**	**C_2_ Legal certaint**	**C_3_ Transparen**	**C_4_ Fairn.& Equity**	**C_5_ Sustainab. use**	**C_6_ Cost-effec tiveness**	**C_7_ Scientific research**	**C_8_ Strength en IPLCs**	**C_9_ Te.transf & coop.**	**C_10_ Incent. biodiv.**	**C_11_ Inno. sol transbou**
**DP1: Material Scope**											
A. *In situ* access	++	++	++	++	+	++	++	0	0	+	+
B. *In situ* + *ex situ* access	++	++	++	++	+	++	+	+	0	++	+
C. *In situ*, *ex situ* + for DSI	+	+	0	−−	−−	−−	−−	0	0	0	0
**DP2: Temporal Scope**											
A. Access for sampling	+	+	+	−	0	−−	−−	0	0	0	0
B. Access for utilization	++	++	++	++	++	++	++	++	++	++	++
C. Access to previously obtained GR for new utiliz.	++	++	++	++	++	++	++	++	++	++	++
**DP3: Utilization Scope**											
A. Research	++	++	+	+	+	−	−−	+	+	+	0
B. Development	++	++	++	+	0	++	++	0	0	0	0
C. Res. & Development	0	+	+	+	0	0	−	+	+	+	0
**DP4: Conditions of access (mandatory BSA)**											
A. Yes	++	++	++	++	++	−	0	+	+	+	0
B. No	++	++	++	+	+	0	0	0	0	0	0
**DP5: Regulatory mechanism**											
A. Notification-based access	+	0	+	+	0	++	++	0	0	0	0
B. Permit-based access	++	++	++	+	+	0	+	+	+	0	0
**DP6: Granting authority**											
A. Centralized single instit.	++	++	++	++	0	++	0	0	0	0	0
B. Several instit. mandated according to type of GR	0	+	+	+	0	−	0	0	0	0	0
**DP7: Standardization**											
A. Standardized	++	++	++	0	0	++	0	0	0	0	0
B. Case by case	++	++	++	++	0	+	0	0	0	0	0
**DP8: Mandatory Local partner**											
A. Yes	+	+	+	+	0	−−	0	+	+	+	0
B. No	++	++	++	++	+	++	0	0	0	0	0
**DP9: Facilitated access for non-commercial research**											
A. Yes	++	++	++	++	+	++	0	0	0	0	0
B. No	+	+	+	0	0	0	0	0	0	0	0
**DP10: Renegotiability**											
A. When user changes	++	++	++	++	0	+	+	0	0	0	0
B. When intent changes	−	−	−	−−	0	0	−	0	0	0	0

##### Calculation of the overall relative priorities of design parameters per stakeholder

**FIGURE 2:**
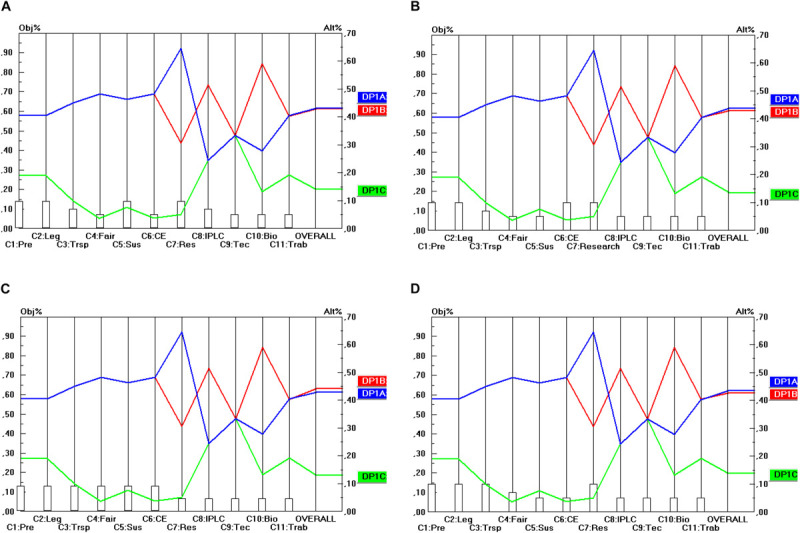
Prioritization of DP_1_ options (Material scope). **(A)** Industrial user point of view, **(B)** academic user point of view, **(C)** provider point of view, **(D)** collections point of view.

**FIGURE 3:**
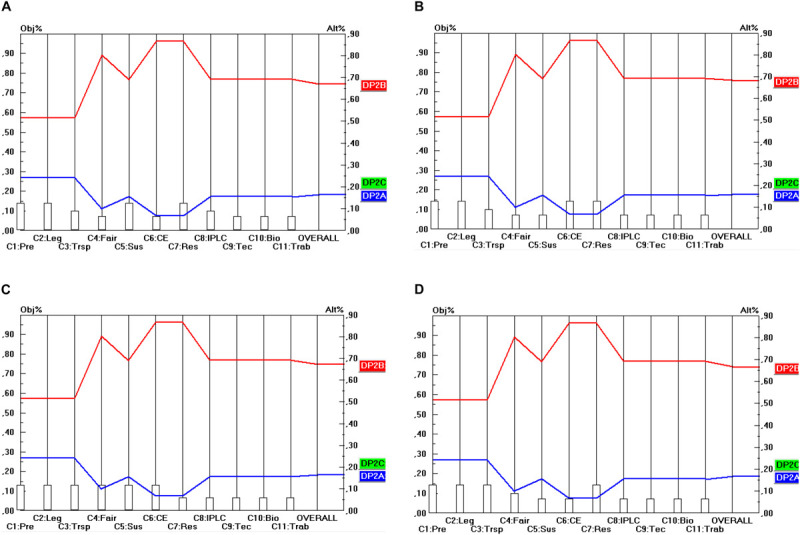
Prioritization of DP_2_ options (Temporal scope). **(A)** Industrial user point of view, **(B)** academic user point of view, **(C)** provider point of view, **(D)** collections point of view.

**FIGURE 4:**
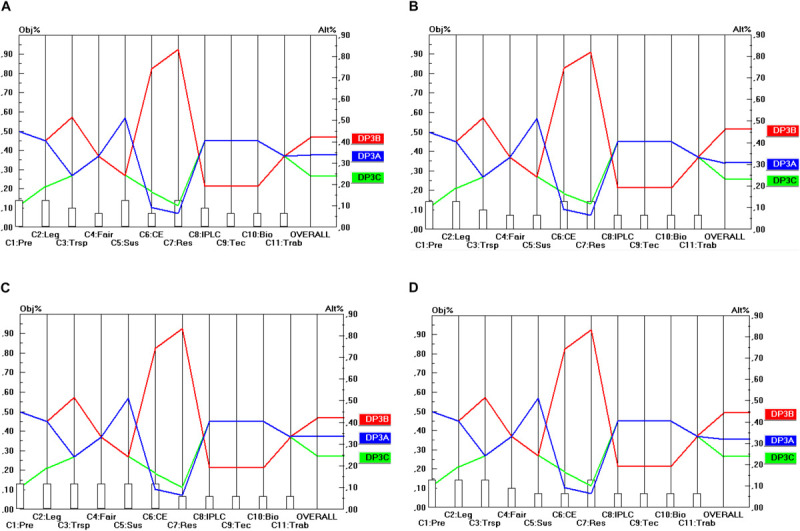
Prioritization of DP_3_ options (Utilization scope). **(A)** Industrial user point of view, **(B)** academic user point of view, **(C)** provider point of view, **(D)** collections point of view.

**FIGURE 5:**
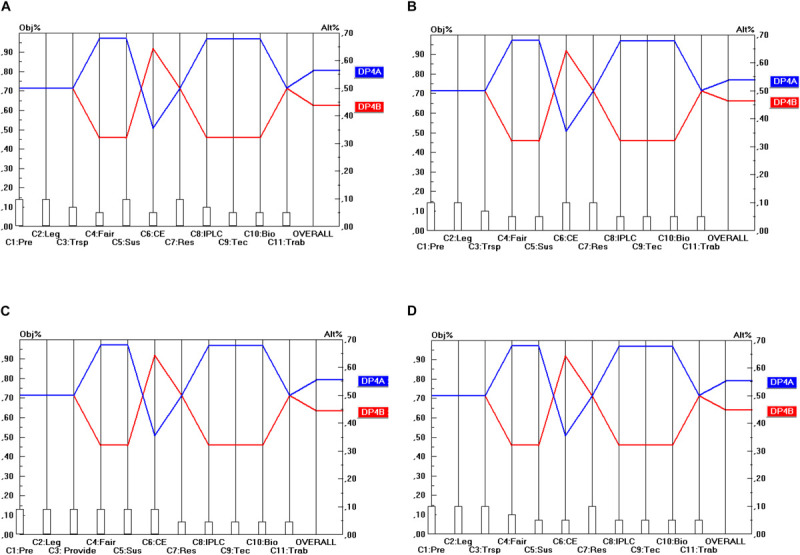
Prioritization of DP_4_ options (Conditions of access). **(A)** Industrial user point of view, **(B)** academic user point of view, **(C)** provider point of view, **(D)** collections point of view.

**FIGURE 6:**
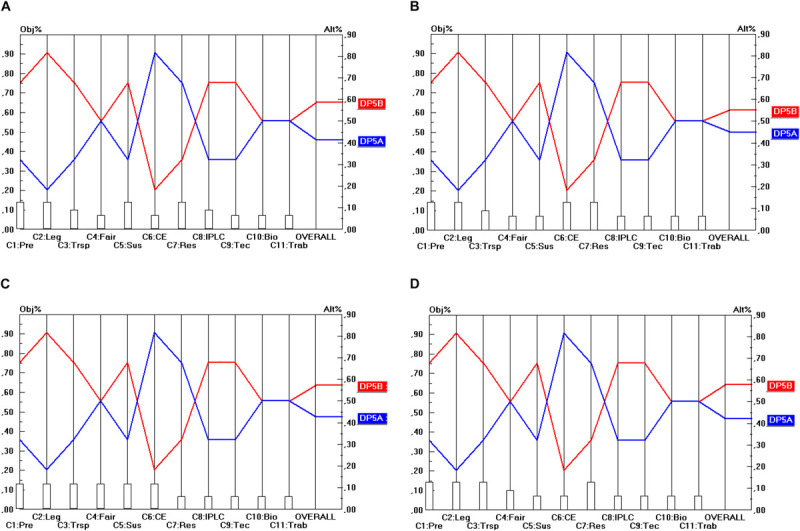
Prioritization of DP_5_ options (Regulatory mechanism). **(A)** Industrial user point of view, **(B)** academic user point of view, **(C)** provider point of view, **(D)** collections point of view.

**FIGURE 7:**
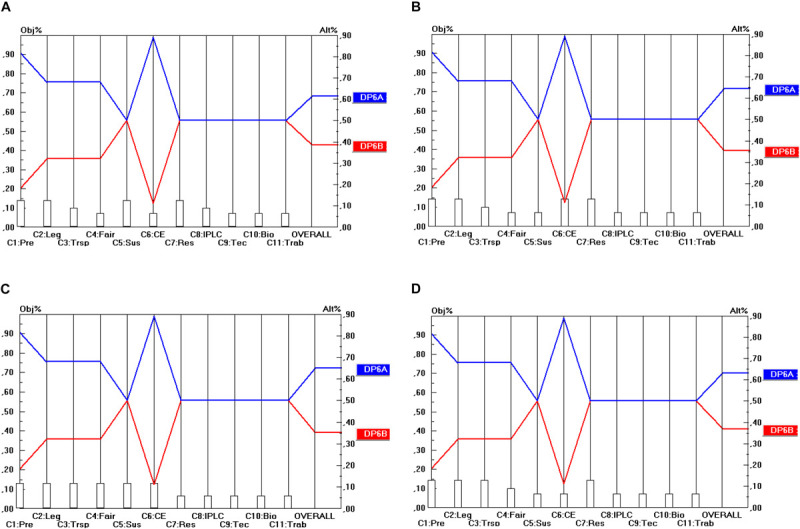
Prioritization of DP_6_ options (Granting authority). **(A)** Industrial user point of view, **(B)** academic user point of view, **(C)** provider point of view, **(D)** collections point of view.

**FIGURE 8:**
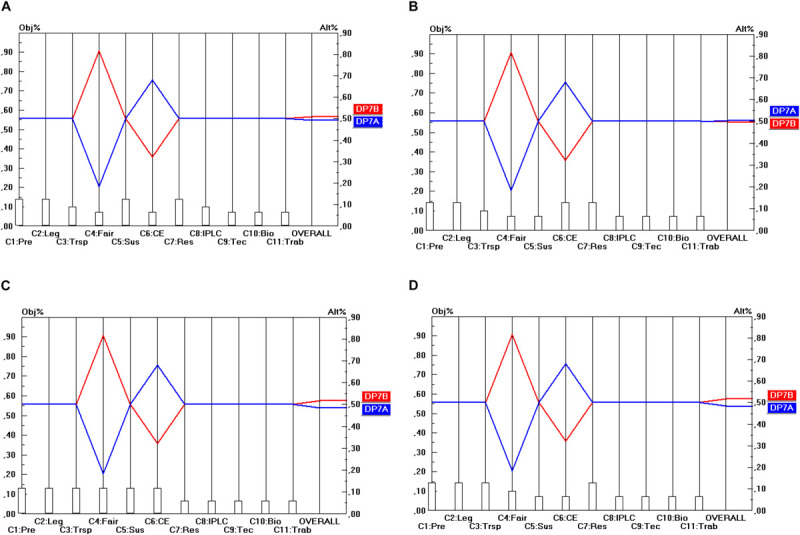
Prioritization of DP_7_ options (Standardization). **(A)** Industrial user point of view, **(B)** academic user point of view, **(C)** provider point of view, **(D)** collections point of view.

**FIGURE 9:**
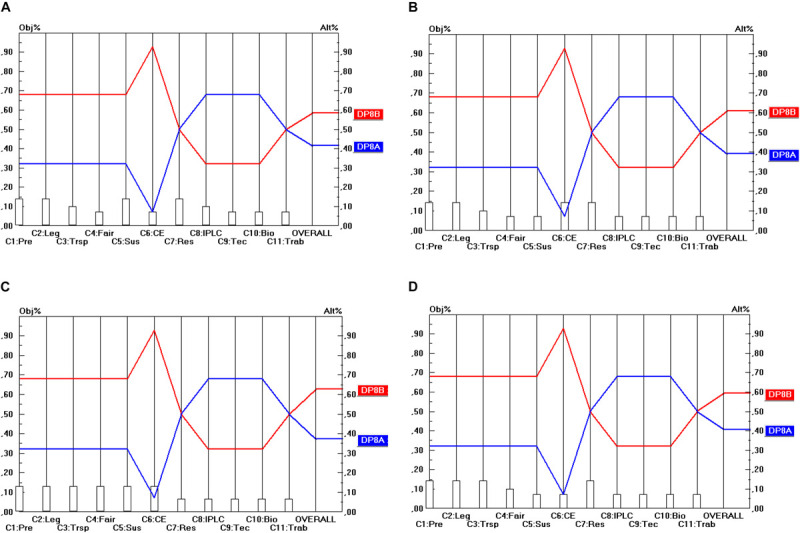
Prioritization of DP_8_ options (Mandatory local partner). **(A)** Industrial user point of view, **(B)** academic user point of view, **(C)** provider point of view, **(D)** collections point of view.

**FIGURE 10:**
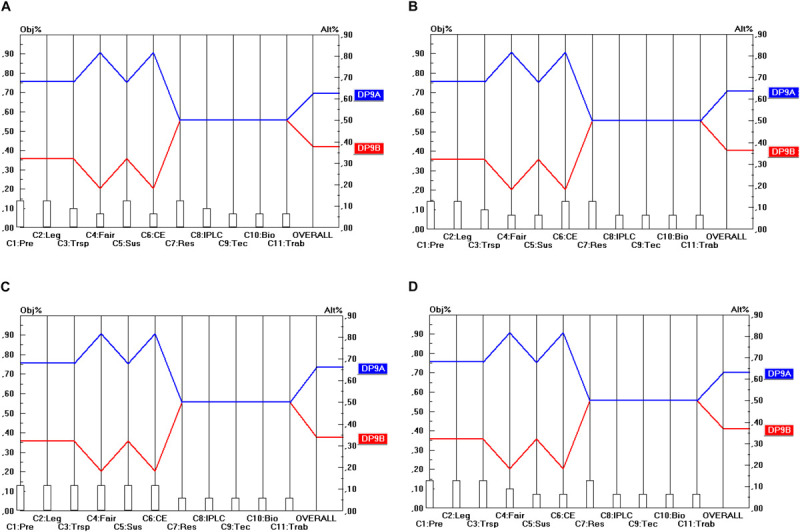
Prioritization of DP_9_ options (Facilitated access for non-commercial research). **(A)** Industrial user point of view, **(B)** academic user point of view, **(C)** provider point of view, **(D)** collections point of view.

**FIGURE 11:**
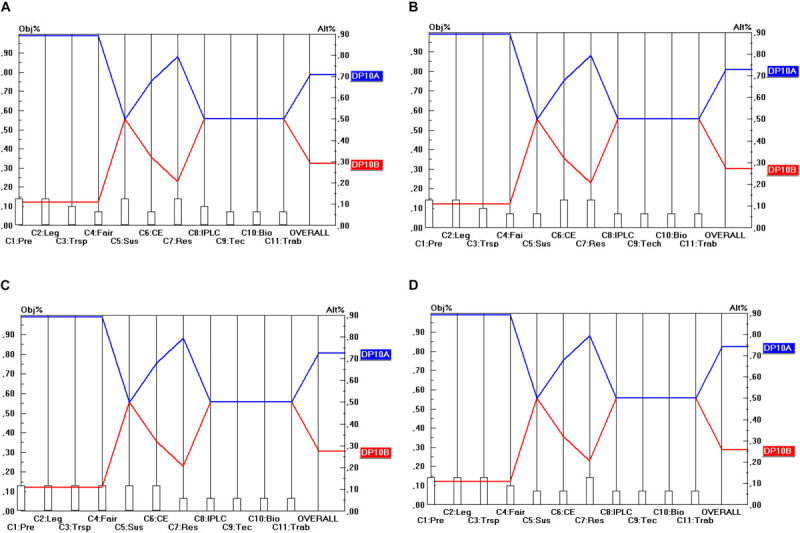
Prioritization of DP_10_ options (Renegotiability). **(A)** Industrial user point of view, **(B)** academic user point of view, **(C)** provider point of view, **(D)** collections point of view.

The final step of the prioritization procedures is to calculate the relative priorities of the final options for the design parameters in terms of their contribution to the stakeholder objectives. This will be done in the manner explained in section “The Use of the Pairwise comparison Mechanism in AHP (in General)” (and according to Eq. 1). Here, two steps can be identified. The first step comprises the prioritization of the different options for the design parameters (DP) in terms of the 11 criteria identified. For instance, regarding DP_1_ (Material scope), there are three options, namely: “*in situ* access” (DP_1__A_), “*in situ* and *ex situ* access” (DP_1__B_) and “*in situ*, *ex situ*, and for DSI” (DP_1__B_) and these three options need to be prioritized in terms of their contribution to the 11 criteria identified (C_1_…C_11_). The same exercise needs to be done for the options (A, B, …) within the remaining DP (DP_2_…DP_10_) and for all of the 11 criteria. For instance, also DP_2__A_, DP_2__B_ and DP_2__C_ need to be compared in terms of their contribution to the 11 criteria. As far as the prioritization of the DP options in terms of the 11 criteria is concerned, the prioritization is based on the (ordinal) performance levels (score categories) of the options, i.e., the five-point ordinal scale (++/+/0:−/−−) subsequently transformed into ratio scales according to the same method as explained in section “General” (Table 3B). The results of these can be read off from Figures 2–11 (as will be explained below).

The second step is to conduct an analysis from each separate stakeholder’s point of view. This means that the different DP options need to be prioritized in terms of their contribution to the overall objectives of each stakeholder group, as measured by the stakeholder criteria (and their associated weights). More concretely, this means that the prioritizations of the DP options need to be combined with the criterion priorities or weights as derived in section “Building Consensus Among Stakeholder Representatives’ Scorings” and shown in Table 5.

The results are shown in Figures 2–11 for the 10 DP studied in this report, for each of the four single stakeholder group’s point of view separately (industrial users, academic users, collections and provider states). For instance, Figure 2 shows the priorities of the three options for DP_1_ (Material Scope), namely: “*In situ* access only” (DP_1__A_), “*In situ* and *ex situ* access” (DP_1__B_) and “*In situ*, *ex situ*, and access for DSI” (DP_1__C_). The 11 criteria (C_1_…C_11_) are represented on the horizontal axis, and their weights are visualized by the height of the vertical bars starting at the criterion’s name. The value of these weights is to be read off from the left vertical axis. For instance, the criterion Predictability (C_1_) has a weight (or priority) of 0.129. The (colored) lines from left to right represent the alternatives (or here: the DP options: DP_1__A_, DP_1__B_, DP_1__C_). The intersection between these (colored) lines from left to right and the vertical lines starting at the criterion’s name represent the priority (i.e., the score) of that alternative (c.q. DP option) on that specific criterion. The value of this priority is to be read off from the right vertical axis. For instance, DP_1__A_ (and also DP_1__B_) obtain a priority of 0.405 on the criterion Predictability (C_1_). Finally, the priority of the DP options in terms of all the 11 criteria taken together (and weighted) are given by the intersection of the (colored) lines from left to right with the right vertical axis. For instance, DP_1__A_ (“*in situ* access only”) obtains a score of 0.430 (in Part A of Figure 2). This score represents the overall relative priority of DP_1__A_ but only in terms of the industrial user’s perspective. A similar approach is followed from the other stakeholder groups’ perspective (shown in Part B, C and D of Figure 2). A particular feature of the analysis of the DP options for the design of ABS frameworks here is that the criteria that are considered relevant by the stakeholder groups are the same for all the four stakeholder groups. This also implies that the scores (priorities) of the DP options will be the same for a particular criterion, irrespective of the point of view from which this criterion is used. Only the criteria weights (as visualized by the height of the vertical bars starting at the criteria’s name) differ among the stakeholder groups and of course also the scores that the DP options obtain on the different criteria differ from the same stakeholder point of view (but not across stakeholder points of view). A consequence of this peculiarity is that the differences between the four parts (A, B, C, D) of Figure 2 are rather small, only the intersections of the (colored) lines from left to right with the right vertical axis differ. In other words, only the overall relative priorities differ between the stakeholder points of view, because the criterion weight sets differ between the stakeholders.

Consequently, the level of conflict between the stakeholder priorities will be relatively low. Regarding ABS systems, the four stakeholders consider the same group of criteria as relevant to them, but to a different extent (i.e., with different weights). This finding may be peculiar to the access component of ABS agreements to GR, but it is doubtful whether this is the case for benefit-sharing agreements. Here, one might expect that the degrees of conflict between stakeholders will be higher.

The low level of conflict between the stakeholders is because, in essence, all of the stakeholders strive to have a system that provides them with the clarity, legal certainty and cost-effectiveness they require in order to either make an informed decision (in the case of providers) or obtain access to GR in a lawful and timely manner (Kamau et al., 2010; Greiber et al., 2012; Kohsaka, 2012; Kariyawasam and Tsai, 2018). Secondly, all stakeholders share the same criteria set as the criteria derive from international legal documents on ABS that set out the general principles for the national ABS systems. This means that all of the national ABS systems should be designed in a way that will achieve these goals, notwithstanding the fact that some goals may be of less importance for some stakeholders than others. Therefore, if these preferred regulatory options are implemented at the national level, the level of conflict occurring in such a system is expected to be low.

This, nevertheless, is unlikely to be the case concerning benefit-sharing. The data obtained during the previous study (Sirakaya, 2019a) on benefit-sharing based on stakeholder interviews demonstrates that most of the users’ interests clash with those of the providers especially in terms of a higher level of conflict between industrial users and provider countries.

As far as the material scope of an ABS framework (DP_1_) shown in Figure 2, is concerned, none of the stakeholders prefers the DP_1__C_ option (*in situ*, *ex situ* access for DSI). Only in relation to C_8_ (Strengthen IPLC) and C_9_ (Technology transfer and cooperation) do the scores of DP_1__C_ match with these of DP_1__A_ and DP_1__B_. For all the other criteria the scores of DP_1__C_ are lower than those of DP_1__A_ and DP_1__B._ Hence, a relation of “weak dominance” exists between DP_1__A_ and DP_1__B_ on the one hand and DP_1__C_ on the other hand. All stakeholders prefer DP_1__A_ or DP_1__B_ and the difference between these two is very small (from an industrial user point of view both DP options even score the same). Hence, the conclusion here is that the stakeholders prefer ABS frameworks to cover “*in situ* access” (DP_1__A_) or “*in situ* and *ex situ* access” (DP_1__B_). On the other hand, a majority of the stakeholders does not favor DSI being covered by the same obligations as those that are imposed on *in situ* or *ex situ* access. As stated in section “Prioritization of the Design Parameters,” this is because all stakeholders benefit from accessing DSI, it would bear high costs for all stakeholders to subject DSI to the same access conditions as GR.

In the approach described above (and that will also be followed below), the options for the design parameters play an important role in the design of specific types of ABS frameworks to GR. Such an approach relies heavily on what Keeney (1996) categorized as “value-focused thinking,” a process approach focusing on eliciting the decision makers’ values or criteria prior to identifying the alternatives. It is only at the next stage that one proactively attempts to identify concrete actions (here: combinations of specific DP options across DP) that can contribute to these predefined values. The set of alternatives is thus said to be “constructed,” instead of being determined externally. By making values explicit and structuring them, it becomes possible to “expand” the feasible set and consider alternatives that were not there at the beginning of the process and yet appear desirable and feasible within the value structure. The value-focused thinking approach sharply contrasts with the “alternative-focused thinking” approach, whereby alternatives are identified at an early stage in the decision-making process and the focus is on distinguishing and choosing between these alternatives. In that case, the pre-defined set of alternatives fundamentally constrains the evaluation process, because it anchors (or even freezes) the thought process, stifling creativity and innovation.

Regarding the temporal scope of ABS frameworks (DP_2_), presented in Figure 3, a similar degree of unanimity among stakeholder groups exists, but now in favor of the DP_2__B_ option. This option even strongly dominates the two other options (DP_2__A_ and DP_2__C_), which even obtain the same score on all criteria. Hence, the conclusion here is that future ABS frameworks will need to be designed in terms of “access for utilization” (DP_2__B_) and not in terms of “access for sampling” (DP_2__A_) or “access to previously obtained GR for new utilization” (DP_2__C_) to fulfill the objectives of all stakeholder groups.

Regarding the Material scope (DP_3_) presented in Figure 4 the level of conflict between the separate criteria is a little higher, but not when all criteria are taken together (i.e., in the overall view per stakeholder). According to each stakeholder point of view the DP_3__B_ option (“Development”) is the most preferable, followed by the DP_3__A_ (“Research”) and the DP_3__C_ option (“Research and Development”). The good overall score of DP_3__B_ here can largely be attributed to the high score on C_7_, C_6_ and C_3_. Hence, it makes sense to test the sensitivity of the result for variations in the weight of these criteria. A sensitivity analysis revealed that the final score of the DP_3__B_ option is insensitive to (small) changes in the weights of the criteria C_7_, C_6_ and C_3_. Only in the event that the weight of C_7_ was reduced from 0.129 to less than 0.030 (i.e., dividing the weight by a factor 4.3) would DP_3__B_ be outperformed by DP_3__A_ and this outperformance would only be very small and it would occur only in terms of the industrial user point of view (and not in terms of the other stakeholders’ point of view).

Here again, the conclusion is that ABS frameworks should be designed along the lines of the DP_3__B_ option (i.e., focusing on “Development”) in order to be consistent with the objectives of all stakeholder groups.

Regarding the Conditions of access (DP_4_), presented in Figure 5, the level of conflict between the separate criteria is very low. The DP_4__A_ option (which includes mandatory BSA) outperforms the DP_4__B_ option (where BSA is non-mandatory) (or yields at least an equal score) on all separate criteria, except the cost-effectiveness criterion (C_6_). In the overall view per stakeholder (i.e., all criteria taken together within each stakeholder view), the DP_4__A_ option is the preferred option of each stakeholder. The sensitivity of this result is very low. The weight of the C_6_ criterion would hypothetically need to be increased to about 0.318 (and from some stakeholder points of view to an even higher level) before rank reversal would occur. This is an increase by a factor of 2.4 from the academic user point of view; from the other viewpoints, the increase would need to be even higher (e.g., a factor 5.74), which is very unrealistic. Hence one can conclude that the final result is rather insensitive to changes in the weights. In other words, sensitivity is low, which can be interpreted as a benefit.

Here again, the conclusion is that ABS frameworks should be designed along the lines of the DP_4__A_ option (whereby BSA is mandatory) to be consistent with the objectives of all stakeholder groups.

Regarding the type of regulatory mechanism (DP_5_), presented in Figure 6, again, the level of conflict between the separate criteria is quite low. The DP_5__B_ option (i.e., with permit-based access) outperforms the DP_5__A_ option (with notification-based access) or yields at least an equal score on all separate criteria but two. The two so-called “discordant” criteria are C_6_ (Cost-effectiveness) and C_7_ (Scientific Research). On these criteria, the DP_5__A_ option is the preferred option and the score difference is rather high on C_6_ and somewhat lower on C_7_. In terms of the overall view per stakeholder (i.e., all criteria taken together within each stakeholder view), the DP_5__B_ option, however, remains the preferred option of each stakeholder.

The sensitivity of this result is rather low, but not as low as in the case of DP_4_. The weight of the C_6_ criterion would hypothetically need to be multiplied by a factor of 1.88 before rank reversal would occur from the academic user point of view. From the other viewpoints, the hypothetical increase needs even to be stronger for rank reversal to occur (the highest increase being by a factor of 4.26 from the industrial user point of view). As regards the C_7_ criterion, rank reversal would only occur with an increase of the C_7_ weight by a factor of 2.41 from the academic user point of view (or even higher in the case of e.g., the provider point of view, namely by a factor of 6.07).

However, if both the C_6_ and the C_7_ weights were increased simultaneously, rank reversal would occur somewhat more quickly. For instance, from the academic user point of view, an increase of both weights simultaneously by a factor of 1.46 would already result in rank reversal (i.e., DP_5__A_ being preferred over DP_5__B_ instead of the other way around), but from the other points of view an increase by a higher factor is needed to cause rank reversal.

Here again, the conclusion is that ABS frameworks should be designed along the lines of the DP_4__B_ option (i.e., with permit-based access) to be consistent with the objectives of all stakeholder groups.

Regarding the options related to the Granting authority (DP_6_), presented in Figure 7, there is no conflict at all, neither between the separate criteria within each stakeholder perspective, nor between the stakeholder points of view (whereby all criteria are taken together after weighing them). In all cases the DP_6__A_ option (whereby one centralized single institution is responsible for granting the permits) is the preferred option. On 6 of the 11 criteria, however, this option scores the same as the alternative option, but in terms of the stakeholder points of view DP_6__A_ remains in all cases the preferred option.

Regarding Standardization (DP_7_), presented in Figure 8, there is a very small level of conflict between the criteria. For 9 of the 11 criteria DP_7__A_ and DP_7__B_ score the same. For the criterion C_4_ (Fairness and Equity) DP_7__B_ outperforms DP_7__A_ and on criterion C_6_ (Cost-Effectiveness), it is the other way around. Moreover, some level of conflict exists between the stakeholder points of view. All stakeholder points of view favor the DP_7__B_ option (i.e., the case-by-case variant), except the academic user point of view that favors DP_7__A_ (i.e., the standardized variant), but the differences are really very small so that academic users might not have a big problem with the DP_7__B_ option. However, the results are quite sensitive to changes in the weights of C_4_ and C_6_. A sensitivity analysis revealed that small changes in C_4_ and C_6_ may lead to a rank reversal of DP_7__B_ and DP_7__A_, particularly in terms of the academic user point of view. On the other hand, this finding reinforces the argument that academic users may not have a big problem when the DP_7__B_ option would be chosen instead of DP_7__A_ option.

Regarding DP_8_ options (i.e., whether a local partner should be mandatory), DP_8__B_ (i.e., no local partner mandatory) is the preferred option, as shown in Figure 9. There is, however, some conflict between the criteria, but not between the stakeholder points of view. The majority of the criteria support the DP_8__B_ option, but a minority of criteria support the DP_8__A_ option (mandatory local partner), namely C_8_ (strengthen IPLC), C_9_ (Technology transfer) and C_10_ (Incentives for biodiversity). As the weight of these criteria is not very high, a sensitivity analysis may be useful in this case.

Here, the sensitivity analysis revealed that the result is relatively insensitive to a hypothetical increase in the weights of these criteria taken separately (i.e., either C_8_, C_9_ or C_10_). The weight of these criteria would need to be increased more than fourfold (more precisely to at least 0.360 and for some stakeholders even to 0.450), which is very unrealistic, before rank reversal would occur. And when the weights of all these so-called “discordant” criteria (i.e., C_8_, C_9_ and C_10_) were increased simultaneously, then the sensitivity analysis revealed that even for a doubling of these three criterion weights simultaneously, rank reversal still does not occur.

Regarding the DP_9_ options (i.e., whether access should be facilitated for non-commercial research), Figure 10 shows that there is unanimity both in terms of the criteria separately as well as in terms of the stakeholder points of view that access should be facilitated for non-commercial research (i.e., the DP_9__A_ option). The only nuance that should be made here is that both options score the same on 5 of the 11 criteria. Hence, in terms of the separate criteria DP_9__A_ weakly dominates DP_9__B_, but in terms of the stakeholder points of view (i.e., comprising all the criteria aggregated within each single stakeholder group separately), there is strong dominance.

Regarding the DP_10_ options (i.e., whether the agreement should be renegotiable when either user or intent changes) there is, according to Figure 11, again unanimity both in terms of the criteria separately as well as in terms of the stakeholder points of view that the option “when user changes” (i.e., the DP_10__A_ option) is preferable to the option “when intent changes” (i.e., the DP_10__B_ option). Again, the nuance that needs to be made is that both options score the same on 5 of the 11 criteria. Hence, in terms of the separate criteria DP_10__A_ weakly dominates DP_10__B_, but in terms of the stakeholder points of view (i.e., comprising all the criteria aggregated within each single stakeholder group separately), there is strong dominance.

## Conclusion

Table 7 shows the optimal options for the parameters that have been identified for the design of ABS frameworks for GR, following a “value-focused thinking” approach (as defined by Keeney, 1996). The top row of Table 7 shows the 10 design parameters (DP_1_…DP_10_). Consequently, each column corresponds to a separate design parameter and comprises the different possible options (A, B, C) for these design parameters (shown by the rows). The DP options (i.e., the options within each separate DP) that command the highest priority are shaded. For instance, as regards DP_2_ (Temporal scope), it is DP_2__B_ (Access for utilization) that commands the highest priority. When two options have been shaded within the same column, this means that both options are ranked (more or less) the same in terms of all the stakeholder groups. For instance, as regards DP_1_ (Material scope), both DP_1__A_ (*In situ* access) and DP_1__B_ (*In situ* access and *ex situ* access) obtain nearly the same priority and the difference between the stakeholder groups is very low (as shown in Figure 2). For DP_10_ (Renegotiability) both options are non-exclusive, which means that the implementation of one of the options does not exclude the implementation of the other option. As they are ranked the same, one could advise integrating both options into the design of the ABS frameworks to be worked out.

**TABLE 7:** Overview of optimal design parameters for the design of ABS frameworks to GR.

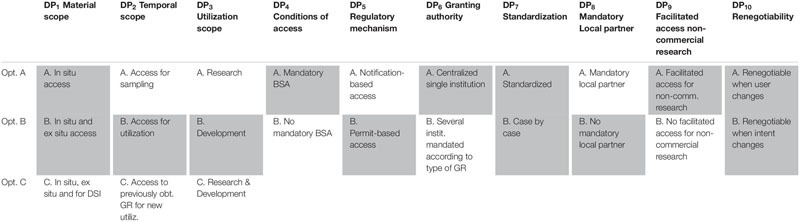

As regards the material scope (DP_1_), the results show that the majority of the stakeholders do not prefer the DSI to be regulated in the same way as GR is, whereas they agree with the regulation of *in situ* and *ex situ* access. Regarding temporal scope (DP_2_), the stakeholders prefer the trigger to be utilization as that would provide them with higher levels of clarity and legal certainty. Especially for provider countries, the cost of monitoring and tracking access would be significantly lower.

Results on DP_3_ demonstrate that the majority of the stakeholders prefer development to be the trigger for access requirements whereas R&D is the least preferred option. This is because the term R&D is not clear-cut and the start of R&D is not apparent in each case.

Regarding DP_4_, the majority prefers mandatory benefit-sharing agreements as the level of legal certainty is much higher for both the users and providers. This is due to the fact that both parties would be able to operate knowing that the activity is in compliance with what the law prescribes. Similar concerns arise in the case of options regarding the granting authority (DP_6_) as both the users and the providers prefer a one-stop shop.

DP_5_ demonstrates a slight difference between the point of view of the industrial and academic users. Whereas the academic users prioritize speed in order to advance in scientific research, industrial users value legal certainty and prefer permits over notifications in order to make sure they have complied with the necessary requirements from the beginning in order to prevent jeopardizing the entire R&D project.

One option the stakeholders did not have a full consensus on is standardization (DP_7_). Many of the stakeholders did prefer standard terms in terms of cost-effectiveness and legal certainty, yet at the same time they found this to have a lower level of fairness in terms of its flexibility, negotiability and adaptability to the situation at hand. Nonetheless, the stakeholders appreciated some level of standardization with the option to tweak the terms to fit a certain situation. The same flexibility is also preferred when it comes to DP_10_ as stakeholders do appreciate permission from the regulatory framework regarding the adaptability of contracts to newly occurring situations such as discovery of a new lead or newly established public-private partnerships.

The sensitivity analysis conducted within DP_8_ demonstrated that all stakeholders prefer the option “no mandatory local partner” in the light of the criteria, including provider countries. This result differs from those displayed under Sirakaya (2019b) where the provider countries preferred the option of imposing mandatory local partners. This may be due to the criteria that play a bigger role in our current research.

DP_9_ portrays a rather straightforward dominance of facilitating access to non-commercial research over non-facilitated access. This is because all stakeholders, including regulators from provider countries see the benefit of research collaborations between the users and provider countries and therefore do want to continue supporting foreign researchers’ interests.

As the level of conflict between the stakeholder priorities was very low in this MCA application, it is not useful to further differentiate the priorities of the DP options according to the stakeholder point of view. Only from the academic point of view was a slightly different ranking of DP options obtained, but the differences were either very small (and often due to the score on one specific criterion) or it occurred only after applying sensitivity analysis (i.e., hypothetically changing the criterion weights).

The reason why the level of conflict between stakeholder preferences is rather low is that the four stakeholder groups consider the same group of criteria to be relevant to them. Only the extent to which these criteria are relevant to them (i.e., the criterion weights) differs between the stakeholders. We assume that this finding may be a peculiar one to ABS frameworks, but that it will no longer be valid for the case of benefit-sharing. Here, one might expect that the degrees of conflict between stakeholders will be higher. Future MCA research, focused on the design of benefit-sharing agreements, will tell us whether this assumption is valid.

The preliminary research (Sirakaya, 2019a) demonstrated that stakeholders significantly differ in their perceptions and preferences toward different benefit-sharing options, although, as displayed in the previous research and solidified with the results of this MCA, stakeholders are much more likely to agree on the commonly used regulatory options on access. This does not mean that the current national ABS frameworks do not result in conflicts. The results of the present research validate that there are options on access that stakeholders can agree on, and if these options are clustered in an ABS framework, the majority of the stakeholders will be able to operate under the law much more smoothly than when the favorable options are placed together with unfavorable ones.

## Data Availability Statement

All datasets presented in this study are included in the article/supplementary material.

## Author Contributions

AS collected the data, the authors designed the methodology together. KD designed the MCA, analyzed the methodology and both the authors contributed to the discussion and conclusions. All authors contributed to the article and approved the submitted version.

## Conflict of Interest

The authors declare that the research was conducted in the absence of any commercial or financial relationships that could be construed as a potential conflict of interest.

## References

[B1] Andersen R. Fauchald O. K. Winge T. Rosendal K. Schei P. J. (2010). *International Agreements and Processes Affecting an International Regime on Access and Benefit Sharing under the Convention on Biological Diversity Implications for its Scope and Possibilities of a Sectoral Approach.* Lysaker: Fridtjof Nansen Institute.

[B2] Bagley M. A. Rai A. K. (2013). The nagoya protocol and synthetic biology research: a look at the potential impacts. *Synbio* 6 1–32.

[B3] Belton V. Stewart T. J. (2002). *Multiple Criteria Decision Analysis: An Integrated Approach.* Boston: Kluwer Academic Publishers 10.1007/978-1-4615-1495-4

[B4] Commons J. R. (1934/1959). *Institutional Economics: Its Place in Political Economy. The.* Madison: University of Wisconsin press.

[B5] Convention on Biological Diversity [CBD] (1992). *United Nations Convention on Biological Diversity, June 5, 1992, 31ILM818, (Entered into Force Dec. 29, 1993).* Brazil, NY: Convention on Biological Diversity 10.1017/S0020782900014728

[B6] Convention on Biological Diversity [CBD] (2011). *ABS Information Kit - English.* Available online at: https://www.cbd.int/abs/information-kit-en/ (Accessed July 24, 2019)

[B7] Coolsaet B. Pitseys J. (2014). Fair and Equitable Negotiations? African Influence and the International Access and Benefit-Sharing Regime. *Glob. Environ. Polit.* 14 82–101. 10.1162/GLEP

[B8] De Brucker K. Macharis C. Verbeke A. (2011). Multi-criteria analysis in transport project evaluation: an institutional approach. *Eur. Transp.* 47 3–24.

[B9] De Brucker K. Macharis C. Verbeke A. (2013). Multi-criteria analysis and the resolution of sustainable development dilemmas: a stakeholder management approach. *Eur. J. Operat. Res.* 224 122–131. 10.1016/j.ejor.2012.02.021

[B10] De Brucker K. Macharis C. Verbeke A. (2015). Two-stage multi-criteria analysis and the future of intelligent transport systems-based safety innovation projects. *IET Intell Trans. Syst.* 9 842–850. 10.1049/iet-its.2014.0247

[B11] De Brucker K. Macharis C. Wiethoff M. Marchau V. (2014). ‘Strategic analysis of stakeholder preferences regarding the design of ITS-based road safety measures’. *IET Intell. Transp. Syst.* 8 190–199. 10.1049/iet-its.2012.0171

[B12] Devi S. Pisupati B. (2018). *Open Source DNA Data, Information and the Nagoya Protocol.* India: FLEDGE.

[B13] Elliott R. Timulak L. (2005). “Descriptive and interpretive approaches to qualitative research,” in *A Handbook of Research Methods for Clinical and Health Psychology*, eds Miles J. Gilbert P. (Oxford: Oxford University Press), 147–157. 10.1093/med:psych/9780198527565.001.0001

[B14] Flach J. dos S. Ribeiro C. van der Waal M. B. van der Waal R. X. Claassen E. (2019). The Nagoya Protocol on Access to Genetic Resources and Benefit Sharing: Best practices for users of Lactic Acid Bacteria. *Pharma Nutr.* 9:100158 10.1016/j.phanu.2019.100158

[B15] Freeman E. R. (1984). *Strategic Management: A Stakeholder Approach.* Boston: Pitman.

[B16] Geary J. Bubela T. (2019). Governance of a global genetic resource commons for non-commercial research: A case-study of the DNA barcode commons. *Int. J. Commons.* 13:205 10.18352/ijc.859

[B17] Gosen S. (2014). *Social Desirability in Survey Research: Can the List Experiment Provide the Truth?* Dissertation, Doktorgrades der Naturwissenschaften, Marburg.

[B18] Greiber T. Moreno S. P. Åhrén M. Nieto J. Chege Kamau E. Oliva M. J. (2012). *An Explanatory Guide to the Nagoya Protocol on Access and Benefit-Sharing.* Gland: IUCN.

[B19] IISD (2019). *Resumed Ninth Meeting of the Ad Hoc Open-ended Working Group to Enhance the Functioning of the Multilateral System of the International Treaty on Plant Genetic Resources for Food and Agriculture: 24-26 October 2019.* Winnipeg: IISD.

[B20] Kamau E. C. Winter G. (2013). An introduction to the international ABS regime and a comment on its transposition by the EU. *LEAD J.* 9 108–126.

[B21] Kamau E. C. Fedder B. Winter G. (2010). The nagoya protocol on access to genetic resources and benefit sharing: what is new and what are the implications for provider and user countries and the scientific community. *Law Environ. Dev. J.* 6:246.

[B22] Kariyawasam K. Tsai M. (2018). Access to genetic resources and benefit sharing: implications of nagoya protocol on providers and users. *J. World Intellect. Prop.* 21 289–305. 10.1111/jwip.12095

[B23] Keeney R. (1996). *Value-Focused Thinking: A Path to Creative Decision Making.* Cambridge, MA: Harvard University Press.

[B24] Koester V. (2012). *The Nagoya Protocol on ABS: Ratification by the EU and its Member States and Implementation Challenges.* Paris: IDDRI.

[B25] Kohsaka R. (2012). *The Negotiating History of the Nagoya Protocol on ABS: Perspective from Japan.* Available online at: http://www.ipaj.org/english_journal/pdf/9-1_Kohsaka.pdf

[B26] Laird S. A. Wynberg R. P. (2018). *). A Fact Finding and Scoping Study on Digital Sequence Information on Genetic Resources in the Context of the Convention on Biological Diversity and Nagoya Protocol.* Brazil, NY: Convention on Biological Diversity.

[B27] Lassen B. (2016). *The Two Worlds of Nagoya: ABS Legislation in the EU and Provider Countries, Discrepancies and how to Deal with them.* Cape Town: Public Eye, Zurich and Natural Justice.

[B28] Lawson C. Rourke M. (2016). Open access DNA, RNA and amino acid sequences: the consequences and solutions for the international regulation of access and benefit sharing. *J. Law Med.* 24 96–118. 10.2139/ssrn.2848136 30136777

[B29] Macharis C. (2005). The importance of stakeholder analysis in freight transport: the MAMCA methodology. *Eur. Trans.* 25–26, 114–126.

[B30] Macharis C. De Witte A. Ampe J. (2007). The multi-actor, multi-criteria analysis methodology (MAMCA) for the evaluation of transport projects: theory and practice. *J. Adv. Transp.* 43 183–202. 10.1002/atr.5670430206

[B31] Morgera E. Tsioumani E. Buck M. (2014). *Unraveling the Nagoya Protocol: Legal Studies on Access and Benefit-sharing. Leiden.* BRILL 10.1163/9789004217188

[B32] Nagoya Protocol (2011). *Access to Genetic Resources and the Fair and Equitable Sharing of Benefits Arising from their Utilization to the Convention on Biological Diversity: Text and Annex.* Montréal: Secretariat of the Convention on Biological Diversity.

[B33] North D. (1990). *Institutions, Institutional Change and Economic History.* New York, NY: Norton 10.1017/CBO9780511808678

[B34] Oberthür S. Rosendal K. (2014). *Global Governance of Genetic Resources: Access and Benefit Sharing after the Nagoya Protocol.* New York, NY: Routledge 10.4324/9780203078020

[B35] Oliva M. J. (2011). Sharing the benefits of biodiversity: a new international protocol and its implications for research and development. *Planta Med.* 77 1221–1227. 10.1055/s-0031-1279978 21674434

[B36] Orsini A. Oberthür S. Po J. (2008). *Transparency in the Governance of Access and Benefit Sharing from Genetic Resources.* Cambridge, MA: MIT Press.

[B37] Overmann J. Scholz A. H. (2016). Microbiological Research Under the Nagoya Protocol: Facts and Fiction. *Trends Microbiol.* 25 85–88. 10.1016/j.tim.2016.11.001 27887771

[B38] Pistorius R. (1997). *Scientists, Plants and Politics: A History of the Plant Genetic Resources Movement.* Rome: International Plant Genetic Resources Institute.

[B39] Prip C. Rosendal K. (2015). *Access to Genetic Resources and Benefit-Sharing from their use (ABS) – State of Implementation and Research Gaps.* Lysaker: FNI. FNI Report 5/2015.

[B40] Roy B. (1996). *Multicriteria Methodology for Decision Aiding.* Dordrecht: Kluwer Academic Publishers 10.1007/978-1-4757-2500-1

[B41] Saaty T. L. (1977). A scaling method for priorities in hierarchical structures. *J. Math. Psychol.* 15 234–281. 10.1016/0022-2496(77)90033-5

[B42] Saaty T. L. (1986). Axiomatic foundation of the analytic hierarchy process. *Manag. Sci.* 7 841–855. 10.1287/mnsc.32.7.841 19642375

[B43] Saaty T. L. (1988). *The Analytic Hierarchy Process.* New York: McGraw-Hill 10.1007/978-3-642-83555-1_5

[B44] Saaty T. L. (1995). *Decision Making for Leaders. The Analytic Hierarchy Process for Decisions in a Complex World.* Pittsburgh: RWS Publications.

[B45] Schei P. J. Tvedt M. W. (2010). *“Genetic Resources” in the CBD: The Wording, the Past, the Present and the Future.* Available online at: https://www.cbd.int/doc/meetings/abs/abswg-09/information/abswg-09-inf-01-en.pdf

[B46] Seiler A. Dutfield G. (2001). *Regulating Access and Benefit Sharing: Basic Issues, Legal Instruments, Policy Proposals.* Available online at: http://www.bfn.de/fileadmin/MDB/documents/access.pdf

[B47] Sirakaya A. (2019a). Balanced options for access and benefit-sharing : stakeholder insights on provider country legislation. *Front. Plant Sci.* 10:1175. 10.3389/fpls.2019.01175 31632420 PMC6781883

[B48] Sirakaya A. (2019b). *Balanced Options for Access and Benefit-Sharing : Stakeholder Insights on Provider Country Legislation.* 1 15. 10.3389/fpls.2019.01175 31632420 PMC6781883

[B49] Swiderska K. (2001). *Stakeholder Participation in Policy on Access to Genetic Resources, Traditional Knowledge and Benefit-Sharing, Case Studies and Recommendations.* London: International Institute for Environment and Development.

[B50] The Economics of Ecosystems and Biodiversity (2010). *Mainstreaming the Economics of Nature: A Synthesis of the Approach, Conclusions and Recommendations of TEEB.* The Economics of Ecosystems and Biodiversity (TEEB), Switzerland.

[B51] UNCTAD (2017). *BioTrade and Access and Benefit Sharing: From Concept to Practice A Handbook for Policymakers and Regulators.* Geneva: UNCTAD.

[B52] Vogel J. H. (2011). The economics of information, studiously ignored in the nagoya protocol on access to genetic resources and benefit sharing. *LEAD J.* 7:52.

[B53] Watanabe M. E. (2015). The nagoya protocol on access and benefit sharing: international treaty poses challenges for biological collections. *Bioscience* 65 543–550. 10.1093/biosci/biv056

[B54] Watanabe M. E. (2019). The nagoya protocol: the conundrum of defining digital sequence information. *Bioscience* 69 480–480. 10.1093/biosci/biz034

[B55] Wynberg R. Laird S. A. (2018). Fast science and sluggish policy: the herculean task of regulating biodiscovery. *Trends Biotechnol.* 36 1–3. 10.1016/j.tibtech.2017.09.002 28964595

[B56] Wyss M. (2017). *Biodiversity and ‘Utilization of Genetic Resources’ in the EU Food Sector.*

